# Degenerative cervical myelopathy presenting as subjective lower limb weakness could be a trap towards misdiagnosis

**DOI:** 10.1038/s41598-020-78139-y

**Published:** 2020-12-03

**Authors:** Chi-An Luo, Meng-Ling Lu, Arun-Kumar Kaliya-Perumal, Lih-Huei Chen, Wen-Jer Chen, Chi-Chien Niu

**Affiliations:** 1 Department of Orthopaedic Surgery, New Taipei Municipal TuCheng Hospital, New Taipei, 236 Taiwan, ROC; 2 Bone and Joint Research Center, Linkou Chang Gung Memorial Hospital and Chang Gung University College of Medicine, Taoyuan, 333 Taiwan, ROC; 3 Department of Orthopaedic Surgery, Spine Division, Kaohsiung Chang Gung Memorial Hospital, Kaohsiung, 833 Taiwan, ROC; 4 Department of Orthopaedic Surgery, Spine Division, Chang Gung Memorial Hospital at Linkou, 5, Fuxing Street, Taoyuan, Guishan 333 Taiwan, ROC; 5 Department of Orthopaedic Surgery, Melmaruvathur Adhiparasakthi Institute of Medical Sciences and Research, Melmaruvathur, Tamil Nadu 603319 India; 6 Department of Orthopaedic Surgery, Chung Shan Hospital, Taipei, 106 Taiwan, ROC

**Keywords:** Neurology, Signs and symptoms

## Abstract

When patients presenting with subjective lower limb weakness (SLLW) are encountered, it is natural to suspect a lumbar pathology and proceed with related clinical examination, investigations and management. However, SLLW could be a sign of degenerative cervical myelopathy (DCM) due to an evolving cord compression. In such circumstances, if symptoms are not correlated to myelopathy at the earliest, there could be potential complications over time. In this study, we intend to analyse the outcomes after surgical management of the cervical or thoracic cord compression in patients with SLLW. Retrospectively, patients who presented to our center during the years 2010–2016 with sole complaint of bilateral SLLW but radiologically diagnosed to have a solitary cervical or thoracic stenosis, or tandem spinal stenosis and underwent surgical decompression procedures were selected. Their clinical presentation was categorised into three types, myelopathy was graded using Nurick’s grading and JOA scoring; in addition, their lower limb functional status was assessed using the lower extremity functional scale (LEFS). Functional recovery following surgery was assessed at 6 weeks, 3 months, 6 months, one year, and two years. Selected patients (n = 24; Age, 56.4 ± 10.1 years; range 32–78 years) had SLLW for a period of 6.4 ± 3.2 months (range 2–13 months). Their preoperative JOA score was 11.3 ± 1.8 (range 7–15), and LEFS was 34.4 ± 7.7 (range 20–46). Radiological evidence of a solitary cervical lesion and tandem spinal stenosis was found in 6 and 18 patients respectively. Patients gradually recovered after surgical decompression with LEFS 59.8 ± 2.7 (range 56–65) at 1 year and JOA score 13.6 ± 2.7 (range − 17 to 100) at 2 years. The recovery rate at final follow up was 47.5%. Our results indicate the importance of clinically suspecting SLLW as an early non-specific sign of DCM to avoid misdiagnosis, especially in patients without conventional upper motor neuron signs. In such cases, surgical management of the cord compression resulted in significant functional recovery and halted the progression towards permanent disability.

## Introduction

Degenerative cervical myelopathy (DCM), occurring due to a mechanical compression of the spinal cord in the neck, is a common spinal dysfunction reported worldwide^1–4^. DCM is often considered a primary differential diagnosis to be ruled out when encountering patients with upper limb symptoms, especially a combination of neck, shoulder and arm pain with hand weakness^5^. However, it is not the case in patients presenting with bilateral subjective lower limb weakness (SLLW). In these patients, it is natural to suspect a lumbar pathology and proceed with related clinical examination, investigations and management. This assumption may not be true when symptoms are inconclusive and misleading^6–8^. There could also be multiple conditions prevailing at one or more sites causing marked cord compression which may cause altered symptoms^9^.

SLLW can be defined as a subjective sense of bilateral lower limb weakness without any objective decrease in lower limb muscle power elicited by manual tests. When patients complain of SLLW, it often means weakness of the entire lower limb with varied difficulty in stepping, but the weakness is not confined to any specific muscle. These patients, if not evaluated appropriately, end up with deficient management, unresolved symptoms and permanent disability^1,10,11^. To overcome this, even though the predominant complaint may be lower limb weakness favouring the possibility of lumbar stenosis, meticulous evaluation to elicit signs of myelopathy and appropriate radiologic screening of the entire spine should be performed to rule out other potential sites of cord compression^12^. This may reveal a solitary compression at the cervical or thoracic region, or tandem spinal stenosis where there is a cervical stenosis in concomitance with stenosis at a lower level^13,14^. Such possibilities should never be ruled out and always be taken into consideration.

Hence, we intend to,

1. Develop an organized algorithm to evaluate and delineate an optimal management strategy for patients presenting with SLLW but having radiological evidence of a solitary cervical or thoracic lesion, or tandem spinal stenosis.
2. Analyse the functional outcomes following decompression surgery in patients with SLLW.

## Methods

After obtaining approval from the institutional review board of Chang Gung Memorial Hospital, Linkou, Taiwan, we retrospectively reviewed data of patients who initially presented to us during the years 2010 – 2016 with bilateral SLLW, but later diagnosed radiologically to have a solitary cervical or thoracic lesion, or tandem spinal stenosis for which definite management in the form of decompression surgery was done. These patients neither presented with radiculopathy along the course of a specific nerve nor neurological deficits due to a compromised nerve, but rather had non-specific symptoms involving both the lower limbs. Only those patients who had such symptoms for a minimum duration of 3 months before our intervention was initiated were selected for the study. Those patients who underwent lumbar surgery elsewhere for the same complaints but did not have any noteworthy betterment were also included in the study. Follow-up data until two years post-surgery was used for interpretation. Those patients with typical signs, including gait changes indicating other neurological problems, those with inconclusive radiological evidence who required a neurologist intervention and those who did not satisfy our minimum follow up requirement were excluded.

**Figure 1:**
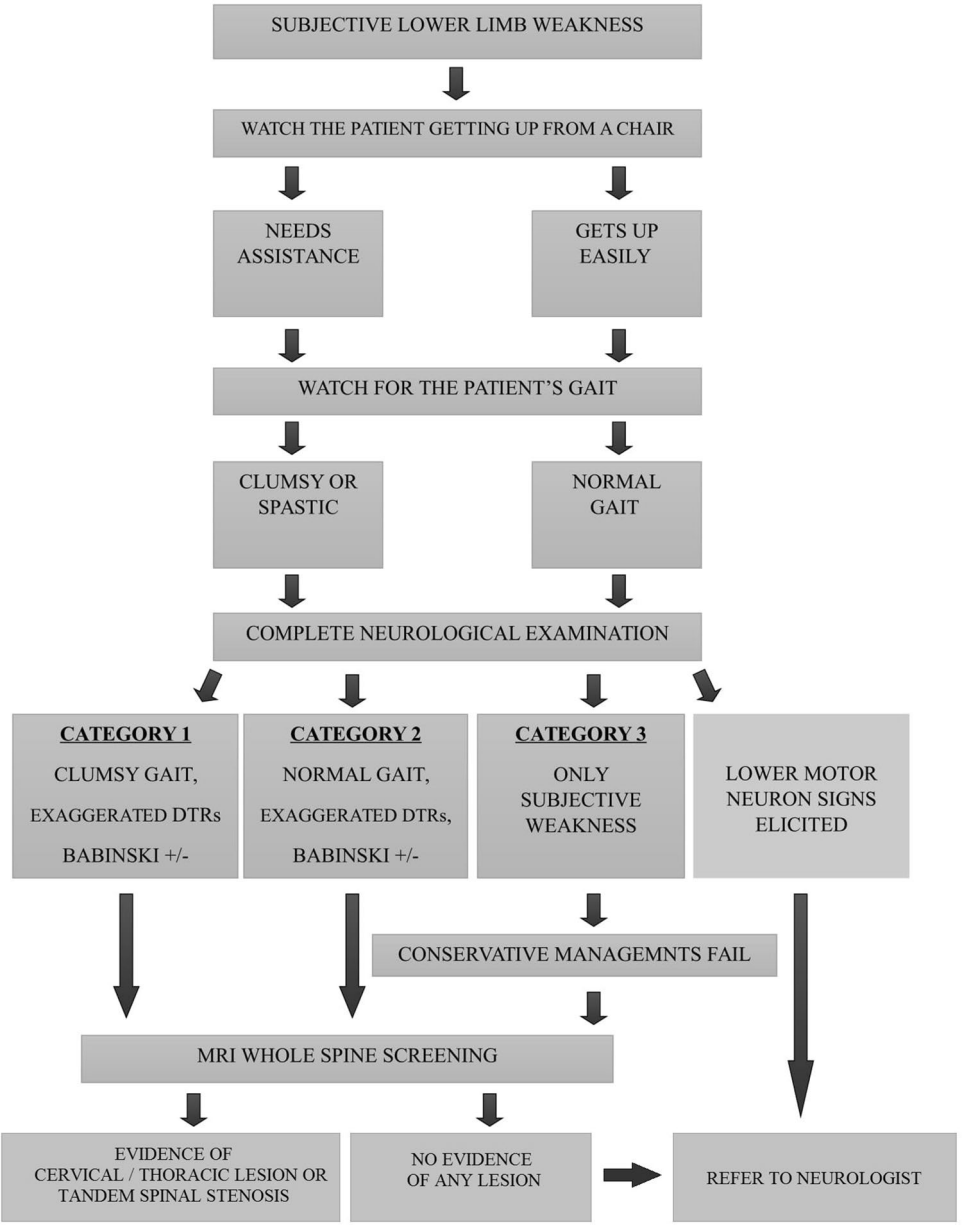
Algorithm for patient evaluation.

The prime complaint that most of the included patients reported was bilateral SLLW. In addition, we observed them having difficulty in getting up from an armless chair. However, on thorough neurological examination as a part of our evaluation protocol (Fig. 1), none of the included patients had specific motor weakness of any grade in the lower limbs or upper limbs; but certain signs indicating myelopathy was elicited. These findings were considered as non-specific presentations of myelopathy due to cord compression. If lower motor neuron signs including muscle atrophy, fasciculations or hypotonia were elicited, those patients were referred to a neurologist for expert intervention and were excluded. As findings were not unanimous, except for SLLW, we divided the patients into three categories based on our observation.

1. Category 1 patients had SLLW, clumsy gait and exaggerated deep tendon reflexes (DTRs) in the lower limbs;
2. Category 2 patients had SLLW, a normal gait but exaggerated DTRs in the lower limbs;
3. Category 3 patients had only SLLW with normal gait and no conventional upper motor neuron (UMN) signs of myelopathy.

Both the Nurick’s grading and JOA score were used to grade myelopathy in the patients. In addition, since the primary complaints were only specific to the lower limbs, functional assessment was done using the lower extremity functional scale (LEFS)^15^. This system is based on a 20-point questionnaire which incorporates physical activity pertaining to the lower limbs like routine household activities, walking, running, etc., and records patient’s response to each question as extreme difficulty, quite a bit of difficulty, moderate difficulty, a little bit of difficulty and no difficulty^15^.

Comprehensive radiological evaluation of the entire spine was performed, which includes appropriate x-rays and magnetic resonance imaging (MRI). Radiological evidence of a spinal cord compressing pathology was identified at the cervical or thoracic region of all patients. Some patients had an additional lumbar lesion along with the cervical or thoracic lesion, causing tandem spinal stenosis. Those patients who had undergone a lumbar decompression surgery considering the radiological evidence of a lumbar lesion, but had persistent symptoms and later diagnosed to have another higher lesion causing myelopathy, were also considered as those with tandem spinal stenosis. Predominantly, the pathology causing stenosis at the cervical level was cervical spondylosis. Some patients had ossification of posterior longitudinal ligament (OPLL) or ossification of ligamentum flavum (OLF) at cervical or thoracic levels. These lesions were found in concomitance with a lumbar herniated intervertebral disc (HIVD) or lumbar spondylosis causing stenosis in those with tandem spinal stenosis.

Considering the potential nature of these cord compressing lesions, surgical decompression of the compressed site was recommended to our patients, rather than to wait for a profound symptom. Appropriate decompression of the spinal cord at the cervical or thoracic compression site was done for all the patients along with stabilization, wherever necessary. All patients were reviewed every month for one year, and once in three months thereafter. Some patients who had tandem spinal stenosis developed fresh radicular symptoms in the lower limbs, which demanded definite management in the form of decompression and stabilization to address the lumbar lesion. Functional outcomes were assessed using LEFS and JOA score following surgery. Functional assessment with LEFS was done at 6 weeks, 3 months, 6 months and 1-year follow-up. Functional assessment with JOA was done at 6 weeks and 2 years follow-up. Patients were followed up for a minimum duration of two years and all necessary data was available in our records. All available data were tabulated for analysis.

Statistical analysis was done using Graph Pad Prism 5 (GraphPad Software Inc., San Diego, CA). Wherever applicable, student’s “T” test and one-way analysis of variance (ANOVA) were used for statistical analysis. Multivariate logistic regression was employed for each potential predictor (binary coded) for final recovery rate of greater than 50%. Odds ratios (ORs) and 95% confidence intervals (CIs) were estimated, and tested using the Wald χ2 test. A probability value of less than 0.05 was considered statistically significant. The study was performed in compliance with the 1964 declaration of Helsinki, its later amendments or comparable ethical standards.

### Ethics approval

This retrospective study with Ref. No. 201700020B0 was approved with informed consent waiver by the Chang Gung Memorial Hospital Institutional Review Board (CGMH IRB, Linkou, Taiwan).

## Results

Our sample (n = 24; Mean age ± standard deviation = 56.4 ± 10.1 years; male = 15; female = 9) was adopted with strict adherence to our selection criteria. All patients had bilateral SLLW for a mean duration of 6.4 ± 3.2 months. Based on our examination protocol, three different types of clinical presentation patterns were observed, and were named as category 1, 2 and 3. The demographic characteristics and location of stenosis as per radiological evidence, in each category were tabulated (Table 1). Category 1 patients (n = 12; 50%) formed the majority of the cohort compared to Category 2 (n = 5; 20.8%) and Category 3 (n = 7; 29.2%).

**Table 1:** Category, demographics and region of stenosis.

Parameter	Category 1	Category 2	Category 3	Statistical significance
Symptom and sign	Subjective weakness Clumsy gait Positive DTRs Babinski + /−	Subjective Weakness Normal gait Positive DTRs Babinski+ /−	Subjective weakness	–
No. of patients	12	5	7	–
Age (years)	54.8 ± 12.43 (32–72)	61.4 ± 9.40 (55–78)	55.6 ± 5.06 (48–64)	*p* = 0.47
Gender	Male = 8; Female = 4	Male = 4; Female = 1	Male = 3; Female = 4	*p* = 0.39
Symptom duration (months)	6.0 ± 3.02 (3–12)	5.0 ± 1.73 (2–6)	8.3 ± 3.97 (3–13)	*p* = 0.17
No. of smoker (%)	3 (25%)	2 (40%)	4 (57%)	*p* = 0.37
Region of stenosis	C-4 TSS (C/T/L) − 2 TSS (C/L) − 6	C-1 TSS (C/T) − 1 TSS (C/L) − 3	C-1 TSS (C/L) − 6	*p* = 0.63
Follow-up (months)	34.9 ± 14.33 (22–60)	41.0 ± 13.71 (24–60)	42.8 ± 13.61 (24–60)	*p* = 0.45

Some values are represented as mean ± standard deviation (range).

*DTR* deep tendon reflex, *C* cervical, *T* thoracic, *L* lumbar, *TSS* tandem spinal stenosis.

**Figure 2:**
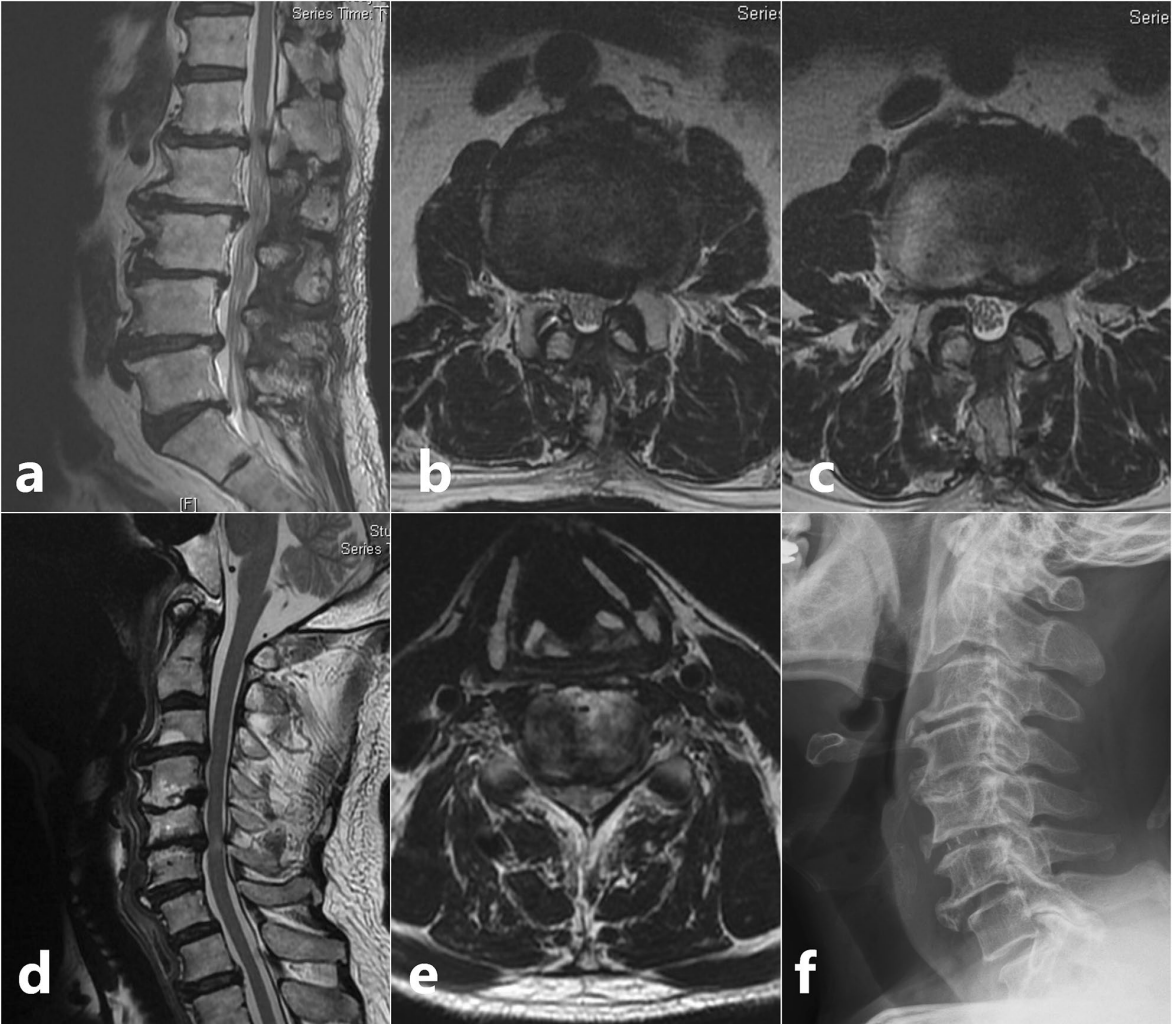
Scenario of a patient who had previous lumbar surgery but later diagnosed to have category 1 signs and radiologic evidence of a cervical pathology and was grouped under Tandem Spinal Stenosis. (**a**) Sagittal T2 weighted MRI of lumbar spine showing degenerative spondylosis; (**b**,**c**) Axial T2 weighted MRI of L2–L3 and L3–L4 levels showing the previous splitting laminectomy done for decompression; (**d**) Sagittal T2 weighted MRI of cervical spine showing degenerative spondylosis extending from C3–C6 levels; Stenosis causing signal intensity changes in the cord at C5–C6 level; (**e**) Axial T2 weighted MRI of C5–C6 level showing apparent stenosis; (**f**) Post ACDF lateral view X-ray image with PEEK cage at C5–C6 level.

Based on Nurick’s grading of myelopathy, 7 patients (29.2%) were grade 1 showing signs of cord compression but had a normal gait, 5 patients (20.8%) were grade 2 who had gait difficulties but fully employed, 12 patients (50.0%) were grade 3 who had gait difficulties preventing employment, but still walked unassisted. Preoperative JOA score (11.3 ± 1.83) revealed only a minimal compromise in function (33.5%); however, functional assessment using LEFS (pre-operative score = 34.4 ± 7.7) indicated a 57% compromise in lower limb function. Whole spine MRI revealed a cervical level cord compression in all the patients, causing degenerative cervical myelopathy. Among whom, the source of compression was cervical spondylosis in 19 patients. The remaining five patients had cord compression due to OPLL. Eighteen patients (75.0%) had tandem spinal stenosis, with MRI findings of cervical spondylosis coupled with thoracic stenosis and/or lower lumbar stenosis; this includes 3 patients (12.5%) having upper thoracic level cord compression due to OLF or OPLL and 7 patients (29.2%) who had previous lumbar surgery before a mean duration of 5.4 months (Fig. 2).

**Figure 3:**
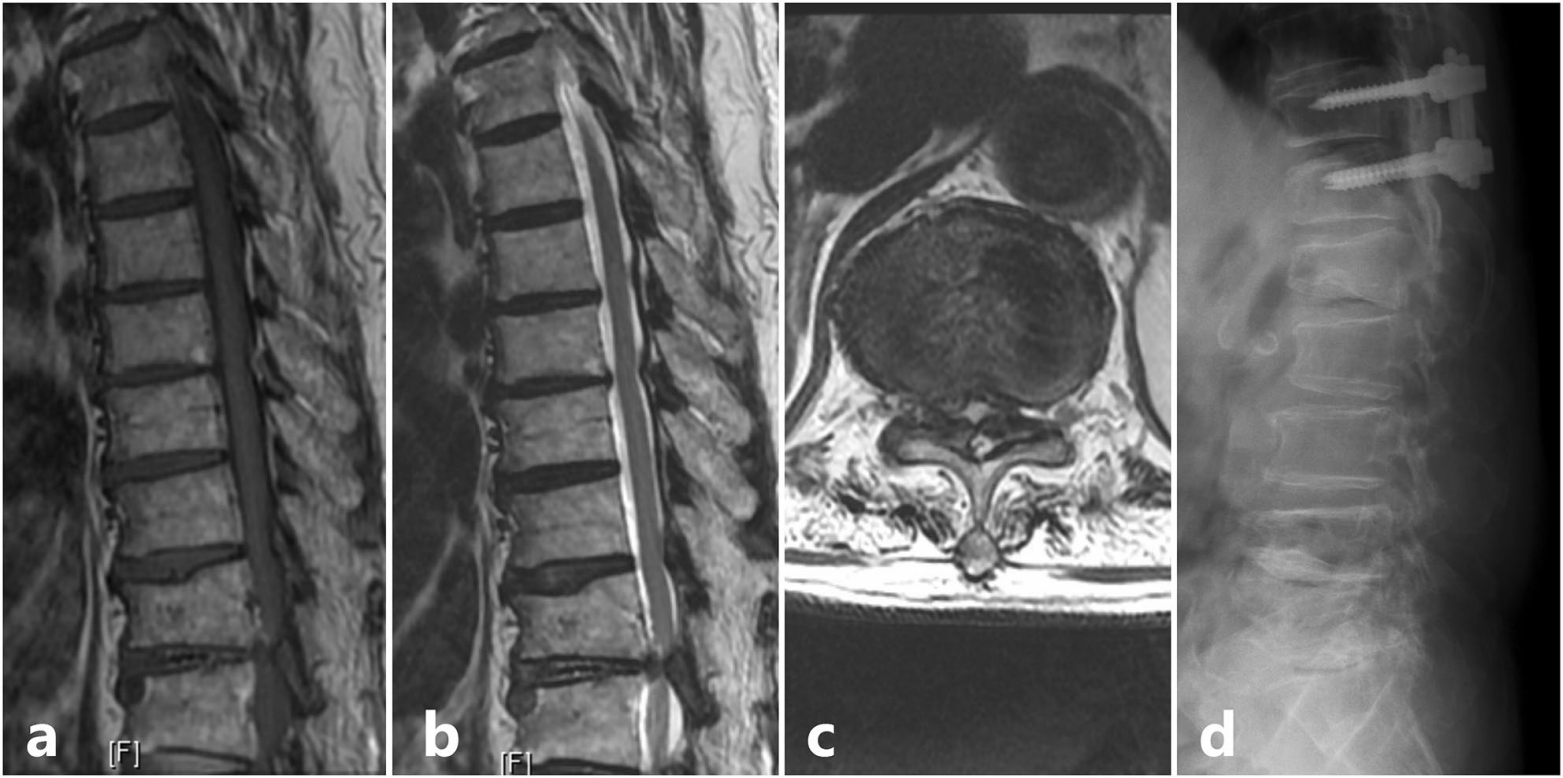
Scenario of a patient with solitary thoracic lesion who presented with category 2 signs; (**a**) Sagittal T1 weighted MRI showing an Ossified Ligamentum Flavum (OLF) at T11-T12 level; (**b**) Sagittal T2 weighted MRI; (**c**) Axial T2 weighted MRI showing the significant stenosis caused due to the OLF at T11-T12 level; (**d**) Post decompression and stabilization lateral view X-ray image.

For cervical lesions, anterior cervical discectomy and fusion (ACDF) was performed for 15 patients, Hirabayashi’s laminoplasty for 5 patients with OPLL, combined ACDF and cervical laminectomy for 1 patient and cervical laminectomy for 3 patients. For thoracic stenosis, 4 patients underwent posterior decompression and stabilization (Fig. 3). Among the 18 patients with tandem spinal stenosis, all were treated with staged surgery. The cervical lesion was treated first with procedures listed above in 10 patients while coexisting thoracic or lumbar lesion were treated first in 8 patients. Patients were on rehabilitation and rest for a period of 6 weeks. After which, they were allowed to carry on with household activities. Four of those patients with tandem spinal stenosis developed radiculopathy and objective lower limb weakness pertaining to the course of a particular nerve after a mean duration of 7.2 ± 1.5 months, which required decompression, interbody fusion and stabilization of the lumbar spine.

Both JOA and LEFS scoring at 6 weeks revealed a modest but statistically significant improvement (JOA score = 13.5 ± 2.34; LEFS score = 39.1 ± 6.4); however, patients were still performing restricted activities and were on further rehabilitation. Functional scoring with LEFS at 3 months showed considerable improvement. On subsequent follow-up, gradual improvement was noticed; all patients were asymptomatic and could perform regular activities by 5.9 ± 4 months. Most patients were at their best possible functional status by 6 months except for those who developed radiculopathy. They were excluded for our functional assessment at 6 months. By the end of one-year, significant improvement was evident compared to the pre-operative status with a mean LEFS of 59.8 ± 2.7 (p < 0.0001). JOA score at two years (13.6 ± 2.76) showed a recovery rate of 47.5% for the entire cohort (Table 2). There was no statistically significant difference between the functional outcomes of each category based on both LEFS and JOA score (Table 3). Follow-up data was available for up to 38.5 ± 13.89 months and none of the patients had any recurrence of similar symptoms during the entire follow-up period. Those patients who underwent lumbar surgery first did not have better outcome until the subsequent cervical and/or thoracic surgery. Multivariate logistic regression showed only preoperative JOA score as a potential predictor (OR 2.77; 95% CI 1.05–7.29) for final recovery rate of more than 50% (Table 4).

**Table 2:** Overall functional outcome based on JOA and LEFS scoring.

Time of assessment	Score	Score	Percentage of functional compromise (%)	Percentage of Improvement in function	Recovery rate by Hirabayashi method	Statistical significance
Preoperative	JOA	11.3 ± 1.83	33.5	–	–	–
	LEFS	34.4 ± 7.7	57	–	–	–
6 weeks	JOA	13.5 ± 2.34	20.6	–	40.8%	*p* < 0.0001
	LEFS	39.1 ± 6.4	51.1	5.9%	–	*p* = 0.01
3 months	LEFS	43.7 ± 7.4	45.4	11.6%	–	*p* < 0.0001
6 months	LEFS	57.4 ± 5.6	28.3	28.7%	–	*p* < 0.0001
One year	LEFS	59.8 ± 2.7	25.3	31.7%	–	*p* < 0.0001
Two years	JOA	13.6 ± 2.76	20.0	–	47.5%	*p* < 0.0001

Some values are represented as mean ± standard deviation (range).

A probability value “*p*” less than 0.05 is considered statistically significant.

Percentage of improvement in function = % of preoperative functional compromise − % of postoperative functional compromise.

Recovery rate by Hirabayashi method (%) = [(postoperative JOA score − preoperative JOA score)/(17 − preoperative JOA score)] × 100.

*LEFS* lower extremity functional scale; *JOA score* Japanese Orthopaedic Association score.

**Table 3:** Functional outcomes among categories.

Time of assessment	Score	Category 1	Category 2	Category 3	Statistical significance
Preoperative	JOA	10.9 ± 2.07	12.0 ± 1.00	11.4 ± 1.90	*p* = 0.54
	LEFS	34.2 ± 8.1	33.2 ± 6.1	35.6 ± 9.1	*p* = 0.88
6 weeks	JOA	12.8 ± 2.59	14.4 ± 0.89	13.9 ± 2.54	*p* = 0.41
	LEFS	40.6 ± 6.2	40.8 ± 4.6	35.4 ± 7.1	*p* = 0.20
3 months	LEFS	43.8 ± 7.3	41.0 ± 4.2	45.6 ± 9.6	p = 0.60
6 months	LEFS	58.9 ± 6.4	57.2 ± 4.9	54.4 ± 3.4	*p* = 0.34
One year	LEFS	59.8 ± 2.1	60.6 ± 3.6	59.1 ± 3.3	*p* = 0.68
Two years	JOA	13.1 ± 2.78	14.4 ± 2.70	14.0 ± 3.00	*p* = 0.63
	RR	43%	50%	53%	*p* = 0.83

*LEFS* Lower Extremity Functional Scale; *JOA* Japanese Orthopaedic Association score.

*RR* Recovery rate (%) = [(postoperative JOA score − preoperative JOA score)/(17 − preoperative JOA score)] × 100; Values are represented as mean ± standard deviation (range).

**Table 4:** Factors predicting an optimal surgical outcome (final recovery rate greater than 50%^16^).

Predictor	Odds ratio	95% Confidence interval	Statistical significance
Gait impairment	1.94	0.19–19.88	*p* = 0.58
Smoking	0.71	0.09–5.50	*p* = 0.74
Age	0.98	0.86–1.09	*p* = 0.59
Symptom duration	0.83	0.59–1.16	*p* = 0.27
Preoperative JOA	2.77	1.05–7.29	*p* = 0.04

Predictors assessed according to the study by AOSpine group^17^.

*JOA* Japanese Orthopaedic Association score.

## Discussion

Degenerative cervical myelopathy involves spinal cord dysfunction from compression rather than other causes such as tumors or vascular lesions^2,4,18^. It is traditionally diagnosed by looking for classical neurologic symptoms and signs including pain and numbness in the extremities, poor coordination, gait imbalance, and bladder problems^12^. Nevertheless, presentations of myelopathy may vary widely depending upon the etiology and stage of the disease. Even though most patients initially present with gait disturbances, classical signs may be absent during early stages^19^. Nagata et al. described that even physical performance decrease could be an early sign of cervical myelopathy that can manifest without the presence of any other classical sign^20^. This was evidenced by measuring the performance of lower extremities such as sit to stand time and one-leg standing time^20^. It is considered that the subtle weakness of lower extremity is beyond the measure limits of clinical evaluation and requires help of an instrument such as a manual dynamometer and/or by doing an electrophysiological study. Given that it is difficult to evaluate such complaints objectively, making a diagnosis of myelopathy demands a high index of suspicion^4^. This was the scenario in most of our patients, where SLLW was the only complaint which affected their daily activities.

Suspicion that symptoms could be due to myelopathy was felt when we observed patients having difficulty in standing up from an armless straight chair. This observation of difficulty to rise up from a chair could be due to myelopathy as described by AT Casey et al.; besides that, this observation constitutes for an integral part of the functional scoring system for cervical myelopathy, otherwise known as the Myelopathy Disability Index^21^. Then, when the patients were made to walk expecting a spastic gait, we noticed a clumsy pace. Following which, a thorough neurological examination was done. Importance was given to DTRs especially knee jerk, ankle clonus and Babinski sign in the lower limbs and Hoffman sign, inverted radial, biceps and triceps reflex in the upper limbs. In addition, we also looked for lower motor neuron signs indicating myopathy or primary muscle disease such as muscle atrophy, fasciculation, hypotonia etc. However, we did not electrophysiologically elicit myelopathy or lower motor neuron deficits in the cervical or lumbosacral myotomes. Several studies have shown that electrophysiological assessment offers a good correlation with the severity of myelopathy and can be a valid predictor of surgical outcomes^22^.

Based on our clinical evaluation, varied presentations were observed; hence, we divided patients into three categories. Category 1 patients were without doubt the classical presentation of myelopathy. Even though the patients of the other two categories demonstrated a relatively normal gait, some subtle differences in gait cycle may be noticed due to myelopathy. Haddas et al.showed that many key muscles take longer to fully contract in the gait cycle including semitendinosus, tibialis anterior and erector spinae, although the onset of their muscle activity is not delayed^23^. Given the possibility of subtle signs, probability of cord compression was not ruled out. Before initiating treatment in such patients, the underlying condition should be diagnosed. MRI plays a key role in diagnosing cord compression^24^; however, considering lower limb weakness, screening should not be restricted to lumbar spine alone but rather include the whole spine. Adhering to our algorithm, a surprisingly high rate of patients in all categories had cord compressing pathologies which was confirmed radiologically by MRI. This indispensable role of MRI whole spine screening should be utilized, especially to diagnose tandem spinal stenosis^25,26^.

Decompressive surgery for degenerative cervical myelopathy is traditionally recommended for patients with moderate and severe grades of myelopathy^1,2,4,13,18^. For patients with mild myelopathy, such as the category 3 patients in our series, the rationale for surgery is as follows: firstly, since their clinical course is trivial, meticulous monitoring is indicated and conservative management must be carried out intensively to be effective^27^. Second, the duration of symptoms is an important factor to consider in the treatment plan for these patients; because, the longer the duration of mild symptoms, the patients tend to get adapted which results in diminished disability^18,27,28^. In our cohort, the mean duration of symptoms for category 3 patients was 8.3 months, the longest among the 3 categories. Even though the indication for surgery versus conservative management for mild myelopathy remains controversial, prioritization of surgical management of the cervical and/or thoracic lesions in category 3 patients demonstrated similar efficacy as that of the other 2 categories (Table 3). This management policy is similar to that of Aydogan et al., who performed staged surgery considering the predominant signs which the patients present with^13^. Almost all patients had resolution of symptoms except for four patients among those with tandem spinal stenosis. They developed fresh radicular symptoms due to the lumbar lesion late after initial surgery; however, myelopathy symptoms had been halted. Hence, lumbar decompression, interbody fusion and stabilisation were performed. Our findings emphasize the need to prioritize definite management of the cervical and/or thoracic cord compressing pathology in selective patients with non-specific symptoms of myelopathy, or SLLW.

Certain confusion arises when a patient with non-specific symptoms as mentioned has a radiological evidence of a lumbar pathology; especially in patients alike our category 3 inclusions. Considering the prevalence of abnormal MRI findings of lumbar spine among asymptomatic subjects, a hastened lumbar surgery should be avoided in patients with inconclusive presentations^29,30^. The possibility of tandem spinal stenosis should always be considered, and patients should be appropriately evaluated. If a lumbar stenosis is identified in concomitance to a cervical or thoracic stenosis, but the patient only presents with SLLW, then the lumbar pathology, especially if below L3, may not be correlated with the symptoms. In such patients with bilateral SLLW, if a lumbar surgery alone was done, they would remain symptomatic or their condition would progress to moderate or severe myelopathy^31^. This was the scenario in 7 of our patients in whom the initial lumbar surgery, performed elsewhere, did not restore normality and patients had continued symptoms that demanded a cervical and/or thoracic decompression.

In our series, the key finding is that, SLLW improved following treatment for DCM presenting with or without conventional upper motor neuron (UMN) signs. The resolution of symptoms can be confirmed by sequential assessment of functional status using LEFS and JOA score. The recovery rates were 43%, 50%, and 53% for category 1, 2, and 3 respectively. The difference in outcome between each category may be related to the baseline JOA score and impairment of gait, similar to the prognostic factors described in the large prospective study on DCM published by the AOSpine group^17^. It should be noted that an optimal surgical outcome (final recovery rate of greater than 50%)^16^ can be satisfactorily achieved in both category 2 and 3 patients, reflecting the importance of clinically suspecting SLLW as a non-specific sign of DCM despite the lack of conventional UMN signs.

### Limitations

First, being a retrospective observational study there are certain limitations pertaining to our methodology; this includes a small sample size, sample selection, and absence of a control group; hence, our analysis may be underpowered to draw potential conclusions. However, our findings suggest that SLLW improved following treatment for myelopathy in patients with or without conventional UMN signs. Second, we used the LEFS for functional assessment as our patients only had complaints of lower limb dysfunction^15,32^. Even though LEFS is not validated for use in DCM, it specifically measures lower limb function which is difficult to be assessed using systems such as JOA scoring. Third, as we did not electro-physiologically evaluate lower motor neuron deficits, we may have missed one or more vital findings which could have biased our decision for surgery. Fourth, the patients with lower motor neuron signs, who were referred to neurologists, were later diagnosed with malnutrition, chronic fatigue syndrome, myasthenia gravis, electrolyte imbalances or thyroid dysfunction but were not followed up further to confirm if DCM co-existed. Given the limitations of this study, further prospective randomised controlled trials are necessary, preferably with a large sample size to aid in factual understanding of diagnosing and managing inconclusive presentations of myelopathy.

## Conclusion

Our findings indicate the importance of clinically suspecting SLLW as an early non-specific sign of DCM to avoid misdiagnosis or delayed diagnosis, especially in those without conventional upper motor neuron signs. Meticulous clinical evaluation and whole spine radiological screening hold the key for diagnosing the underlying disorder. Our results indicate the need to prioritize definite management in the form of surgery for the cervical and/or thoracic cord compressing pathology in selective patients with SLLW to resolve symptoms of lower limb weakness and to prevent progression to established myelopathy.

## Supplementary information

Supplementary Information.

## Data Availability

All data generated or analyzed during this study will be made available by the corresponding author on reasonable request.
