# Supplementary material for: Degenerative cervical myelopathy presenting as subjective lower limb weakness could be a trap towards misdiagnosis
Source: Sci Rep. 2020 Dec 3;10:21188. doi: 10.1038/s41598-020-78139-y (PMC7712653; doi:10.1038/s41598-020-78139-y)
Supplement: Supplementary file 1 — Supplementary Information. [file 41598_2020_78139_MOESM1_ESM.pdf]

Degenerative Cervical Myelopathy Presenting as Subjective Lower Limb Weakness Could be a Trap Towards Misdiagnosis

Authors: Chi-An Luo, Meng-Ling Lu, Arun-Kumar Kaliya-Perumal, Lih-Huei Chen, Wen-Jer Chen, Chi-Chien Niu

Case	Age	Sex	Smoke	Category	S/S duration (month)	Diagnosis	Region	OP 1	OP 2	Nurick preop	Nurick postop 2y	JOA preop	JOA postop 6w	JOA postop 2y	Recovery rate 2y	LEFS preop	LEFS postop 6w	LEFS postop 3m	LEFS postop 6m	LEFS postop 1y	Duration from OP to improvement (month)	Follow-up (month)
1	48	M	N	1	12	DCM, C3-6	CS	ACDF	nil	3	2	9	12	12	38	20	27	36	64	57	4.4	39
2	59	M	Y	3	13	DCM, C4-T1	CS	ACDF	nil	2	2	13	13	14	25	27	27	40	53	56	12.0	24
3	64	M	Y	3	12	1.DCM, C3-7<br>2.LS, L3-5	TSS (CS/LS)	ACDF	Lumbar laminectomy	1	1	8	9	8	0	40	40	40	52	58	6.0	36
4	61	F	N	1	3	DCM, C3-6	CS	ACDF	nil	3	2	12	16	14	40	34	44	36	70	60	12.0	24
5	58	M	Y	1	5	DCM, OPLL, C3-7	CS	Cervical LMP	nil	3	2	12	14	15	60	35	45	40	61	60	4.2	24
6	32	M	N	1	6	1.DCM, C3-6<br>2.DLS L4-5	TSS (CS/LS)	Lumbar PLF	PCF	3	1	15	15	17	100	38	40	44	58	60	12.0	22
7	55	M	Y	2	2	1. DCM, OPLL, C3-5.<br>2. OYL of T3,4,9,10	TSS (CS/TS)	Cervical LMP	Thoracic Laminectomy	2	1	12	14	17	100	27	40	46	59	65	12.0	39
8	43	F	N	1	12	1.DCM, OPLL, C4~6,<br>2.OPLL, T2-3, T6-8<br>3.DLS, L5S1	TSS (CS/TS/LS)	Cervical LMP	Cervcial/Thoracic Laminectomy	3	3	10	10	11	14	40	44	44	64	61	6.0	60
9	71	M	N	1	4	1. DCM, C6-T1<br>2. DLS, L3-5	TSS (CS/LS)	Cervical laminectomy+ACDF	Lumbar PLF/TLIF	3	3	10	9	9	-14	20	46	58	52	63	12.0	48
10	58	M	N	2	5	1. DCM, C4-6<br>2. LS, L3-5	TSS (CS/LS)	ACDF	nil	1	1	13	15	14	25	27	40	44	52	63	2.8	48
11	59	M	Y	2	6	1. DCM, C5-6<br>2. DLS, L3-5	TSS (CS/LS)	ACDF	Lumbar PLF/TLIF	2	3	11	13	10	-17	40	44	35		61	6.0	34
12	57	F	N	2	6	1. DCM, C5-6<br>2. DLS	TSS (CS/LS)	ACDF	nil	1	1	11	15	15	67	38	46	40	62	57	1.7	60
13	56	F	N	3	12	1. DCM, C4-5<br>2. LS, L4-5	TSS (CS/LS)	Lumbar PLF	ACDF	1	1	12	14	16	80	40	34	40	52	57	7.5	48
14	78	M	N	2	6	DCM, C3-5	CS	ACDF	nil	1	1	13	15	16	75	34	34	40	52	57	6.0	24
15	47	M	N	1	3	DCM, C5-7	CS	ACDF	nil	3	2	10	13	14	57	36	40	36	56	59	4.9	60
16	54	M	Y	1	5	1. DCM C3-4<br>2. TS, T12<br>3. LS, L4-S1	TSS (CS/TS/LS)	ACDF	Thoracic laminectomy + Lumbar PLF/TLIF	3	3	10	11	10	0	34	29	44	58	60	6.0	24
17	55	F	N	3	3	1. DCM, C4-6<br>2. DLS, L4-5	TSS (CS/LS)	Lumbar PLF	ACDF	1	1	13	16	16	75	34	27	40	55	64	12.0	60
18	61	F	N	1	6	1. DCM, OPLL, C3-6<br>2. LS, L3-L5	TSS (CS/LS)	Lumbar PLF/TLIF	Cervical LMP	3	3	7	9	9	20	27	44	40	64	63	2.1	40
19	52	F	N	3	6	1. DCM, C5-6<br>2. DLS, L3-4	TSS (CS/LS)	Lumbar PLF	ACDF	2	2	10	14	13	43	20	40	40	60	56	0.4	60
20	72	M	N	1	4	1. DCM, C7-T1<br>2. LS, L3-5	TSS (CS/LS)	Lumbar laminectomy	Cervical laminectomy	3	3	13	16	16	75	40	40	44	52	57	1.7	24
21	43	F	N	1	6	1. DCM, OPLL, C1-5<br>2. TS, OPLL, T5-6, OYL, T3-11	TSS (CS/TS)	PTF	Cervical LMP	3	2	12	15	15	60	40	44	46	49	57	3.3	24
22	55	M	Y	3	6	1. DCM, C3-5<br>2. DLS, L4-5	TSS (CS/LS)	Cervical LMP	lumbar PLF/TLIF	2	2	11	14	14	50	44	46	58		60	2.5	36
23	67	M	Y	1	6	1. DCM, C3-6<br>2. LS, L1-3	TSS (CS/LS)	ACDF	Lumbar laminectomy	3	2	11	14	15	67	46	44	57		61	1.7	30
24	48	F	Y	3	6	1. DCM, C3-7<br>2. DLS, L4-5	TSS (CS/LS)	Lumbar PLF/TLIF	ACDF	1	1	13	17	17	100	44	34	61	61	63	2.8	36

DCM: Degenerative cervi
DLS: Degenerative lumba
OPLL: Ossification of po
OYL: Ossification of yell

CS: Cervical stenc
TS: Thoracic stenc
LS: Lumbar steno
TSS: Tandem spin

LMP: Laminoplasty (open-door)
PLF: Posterolateral fusion
TLIF: Transforaminal lumbar interbody fusion
PCF: Posterior cervical fusion
PTF: Posterior thoracic fusion
